# The pyramidalis–anterior pubic ligament–adductor longus complex (PLAC) and its role with adductor injuries: a new anatomical concept

**DOI:** 10.1007/s00167-017-4688-2

**Published:** 2017-09-02

**Authors:** Ernest Schilders, Srino Bharam, Elan Golan, Alexandra Dimitrakopoulou, Adam Mitchell, Mattias Spaepen, Clive Beggs, Carlton Cooke, Per Holmich

**Affiliations:** 1 Fortius Clinic, 17 Fitzhardinge Street, W1H 6EQ London, UK; 2 School of Sport, Leeds Beckett University, Leeds, West Yorkshire UK; 3 Orthopaedics, Lennox Hill Hospital, New York, NY USA; 4 Mount Sinai School of Medicine, New York, NY USA; 5 Orthopaedics, Maimonides Medical Center, Brooklyn, NY USA; 6 The Wellington Hospital, The London Hip Arthroscopy Centre, London, UK; 7 Radiology, Fortius Clinic, London, UK; 8 Radiology, GZA, Antwerpen, Belgium; 9 Leeds Trinity University, Leeds, West Yorkshire UK; 10 Sports Orthopedic Research Center - Copenhagen (SORC-C), Department of Orthopedic Surgery, Copenhagen University Hospital, Amager-Hvidovre, Copenhagen, Denmark; 11 Aspetar Orthopaedic and Sports Medicine Hospital, Sports Groin Pain Center, Doha, Qatar

**Keywords:** Adductor longus avulsions, Anterior pubic ligament, Pyramidalis–anterior pubic ligament–adductor longus complex (PLAC), Pyramidalis, Groin pain in athletes, Rectus abdominis, Adductor injuries, Groin anatomy

## Abstract

**Purpose:**

Adductor longus injuries are complex. The conflict between views in the recent literature and various nineteenth-century anatomy books regarding symphyseal and perisymphyseal anatomy can lead to difficulties in MRI interpretation and treatment decisions. The aim of the study is to systematically investigate the pyramidalis muscle and its anatomical connections with adductor longus and rectus abdominis, to elucidate injury patterns occurring with adductor avulsions.

**Methods:**

A layered dissection of the soft tissues of the anterior symphyseal area was performed on seven fresh-frozen male cadavers. The dimensions of the pyramidalis muscle were measured and anatomical connections with adductor longus, rectus abdominis and aponeuroses examined.

**Results:**

The pyramidalis is the only abdominal muscle anterior to the pubic bone and was found bilaterally in all specimens. It arises from the pubic crest and anterior pubic ligament and attaches to the linea alba on the medial border. The proximal adductor longus attaches to the pubic crest and anterior pubic ligament. The anterior pubic ligament is also a fascial anchor point connecting the lower anterior abdominal aponeurosis and fascia lata. The rectus abdominis, however, is not attached to the adductor longus; its lateral tendon attaches to the cranial border of the pubis; and its slender internal tendon attaches inferiorly to the symphysis with fascia lata and gracilis.

**Conclusion:**

The study demonstrates a strong direct connection between the pyramidalis muscle and adductor longus tendon via the anterior pubic ligament, and it introduces the new anatomical concept of the pyramidalis–anterior pubic ligament–adductor longus complex (PLAC). Knowledge of these anatomical relationships should be employed to aid in image interpretation and treatment planning with proximal adductor avulsions. In particular, MRI imaging should be employed for all proximal adductor longus avulsions to assess the integrity of the PLAC.

## Introduction

The symphyseal and perisymphyseal area has become of increasing interest for hip surgeons, sports physicians and radiologists dealing with hip impingement and complex pain syndromes of the inguinal/adductor and lower abdominal area. An anatomical explanation has been sought for inguinal, adductor related and pubic pain experienced by athletes [32].

Several anatomy studies have been performed to examine the adductor tendons [11, 30, 31] and the connection with the distal rectus sheath [23, 25, 27]. Some studies suggest that a direct anatomical connection exists between the caudal rectus abdominis muscle and the proximal origin of the adductor longus [6, 14, 19, 20, 34].

It has been the first author’s clinical observation that athletes with traumatic adductor longus avulsions often present with associated unilateral abdominal pain and lower abdominal haematomas. When examined on MRI scans or intraoperatively, it is the first author’s experience that in such patients, it is the pyramidalis muscle, and not the rectus abdominis, that remains attached to the adductor longus preventing caudal and lateral retraction.

If correct, this finding demonstrates that there is a direct anatomical connection between the pyramidalis muscle and the adductor longus muscle, suggesting that the role of the pyramidalis in this type of injury may have been underestimated. The involvement of the pyramidalis in groin pain or pubic-related pain in athletes has not previously been reported. Indeed, in a study reviewing 347 abdominal muscle strains in professional baseball, no injuries to the pyramidalis were reported [7].

There are conflicting views in the literature with regard to the anatomical position of the rectus abdominis: several imaging and anatomy studies [6, 11, 20] reported the rectus abdominis muscle to lie anterior to the pubic symphysis. This description lies in direct contrast to the findings of numerous eighteenth- and nineteenth-century anatomy books [4, 8, 13, 15–17, 22, 29, 33] and one cadaver study in 2007 [25] which found the pyramidalis to be the only abdominal muscle encountered anterior to the pubic symphysis.

The pyramidalis muscles are paired and lie between the anterior surface of the rectus abdominis and the posterior surface of the rectus sheath. The muscle exhibits a mixed fibre-type composition [18]. The function of the pyramidalis muscle is to tension the linea alba [8, 18] and stretch the abdominal aponeurosis [17] to assist the rectus abdominis so as to give greater power to the oblique and transverse muscles [4].

Adductor longus injuries are complex, and MRI interpretation can be difficult. The aim of the study is to systematically investigate and describe the anatomy anterior to the pubic symphysis and pubic bones with special focus on the pyramidalis muscle and its anatomical connections with adductor longus and rectus abdominis, to elucidate injury patterns occurring with adductor avulsions.

The pyramidalis muscle is the only muscle lying anterior to the pubic symphysis and pubic bones. The pyramidalis muscle has a strong direct anatomical connection with the adductor longus. There is no significant connection between the rectus abdominis and the adductor longus.

## Materials and methods

### Dissection technique and observations

Two orthopaedic surgeons (ES, SB) with a vast experience in athletic groin injuries worked jointly to systematically dissect the anterior pubic soft tissues in seven fresh-frozen cadaveric male pelvises [median age 67 years (60–79)]. Standard surgical dissection techniques were used to perform a meticulous layer-by-layer dissection from the skin down to the anterior pubic bone with macroscopic findings discussed and reported. All specimens dissected included the pelvis to mid-diaphyseal femur. The skin and subcutaneous fat were excised from the level of anterior superior iliac spines (ASIS) to mid-thigh exposing the anterior rectus sheath, external oblique fascia and fascia lata. Care was taken to preserve the integrity of the fibres of the individual fascial plains. The linea alba was inspected for anatomical variation (i.e. flat or cord-like).

The fibre orientation of the anterior rectus sheath, external oblique fascia, anterior pubic ligament and fascia lata was noted, with a window subsequently made in the anterior rectus fascia adjacent to the linea alba, allowing access to the deeper fascia anterior to the pyramidalis muscle. The fibre orientation of this fascia was also recorded, with all such fascial fibre orientations noted based on orientation in a frontal plane.

The fascia was then completely excised from the level of ASIS to mid-thigh fully exposing the pyramidalis muscle from superior to inferior, rectus abdominis, adductor longus and pectineus. For each specimen, it was noted if the pyramidalis muscle was present or absent, unilateral or bilateral. The dimensions of the pyramidalis muscle were then measured using an electronic calliper KD Tools 3756: the width of the base of the muscle and the height [top to centre of the base (bissectrice)]. The distances were measured in centimetres, and each measurement was repeated three times. Averages were calculated for the measurements for each individual specimen. Subsequently, the average of the individual averages is calculated to determine mean values for the study group.

The pyramidalis muscle was then sharply detached from the linea alba and folded distally to inspect the posterior surface of the muscle and its attachment to the pubic bone, thus facilitating exposure of the distal part of the rectus abdominis. The anterior rectus abdominis was inspected, along its insertion onto the pubis, with its relationship with other structures noted. Each insertion was defined as either tendinous or muscular. Specific attention was also paid to whether each musculotendinous junction was cranial to the pubic bone.

Subsequently, a scalpel was employed to sharply detach the adductor longus proximally from its attachment with the deep portion of the anterior pubic ligament. The fibrocartilage deep to the adductor longus tendon was also sharply detached from anterior pubic bone caudal to the pubic crest in a manner intended to simulate an avulsion through the fibrocartilage as seen with acute adductor longus avulsions.

Lastly, the external tendon of the rectus abdominis was detached distally to visualize the insertion. The internal tendon of the rectus abdominis was also traced caudally with any encountered connection to the adductor longus or deep portion of the anterior pubic ligament noted. Finally, the portion of the anterior pubic ligament found anterior to symphysis was resected to maximize exposure of the internal tendon of the rectus abdominis.

The study did not represent research with living individuals or the use of protected health information and was declared exempt by our institutional review board. The study was undertaken at Maimonides, Medical Center, Brooklyn, NY, USA.

### Statistical analysis

For each pyramidalis specimen, measurements were taken three times with callipers zeroed between measures. The averages and standard deviations were computed. A paired, two-tailed Wilcoxon test was used to compare dimensions for left- and right-hand sides, with *p* < 0.05 deemed to be significant. The rationale was to assess symmetry of the muscle.

## Results

### Cadaveric dissection

The following anatomical descriptions apply to our findings in all specimens; when variations between the specimens are observed, this will be mentioned separately.

### Abdominal aponeurosis and anterior pubic ligament (superficial portion)

**Fig. 1:**
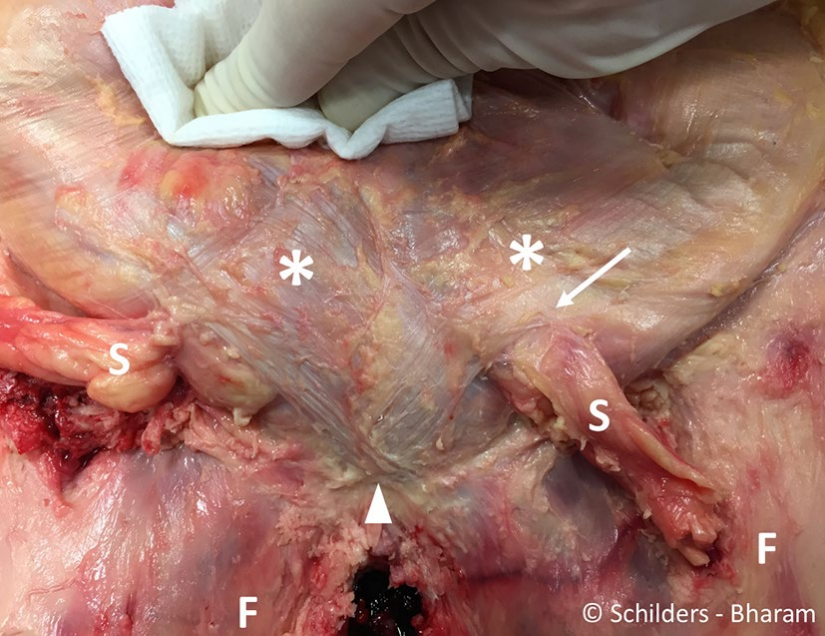
Male cadaver. Removal of the skin and the subcutaneous fat from the level of the anterior superior iliac spines to mid-thigh exposing the aponeurosis of the (*asterisk*) of the abdominal external oblique muscle, the rectus abdominis, the pyramidalis, the superficial part of the anterior pubic ligament (*arrowhead*) and the fascia lata (*F*). *S* spermatic cords. *Arrow* external inguinal ring

The aponeurosis of the external oblique and anterior rectus sheath has obliquely orientated fibres converging onto its insertion at the linea alba. The fibres of the external oblique, which form the medial and superior part of the external inguinal ring, cross anteriorly to the pubic bone to form the superficial layer of the anterior pubic ligament and continue distally in the fascia lata covering the medial thigh and adductors on the contralateral side (Fig. 1).

**Fig. 2:**
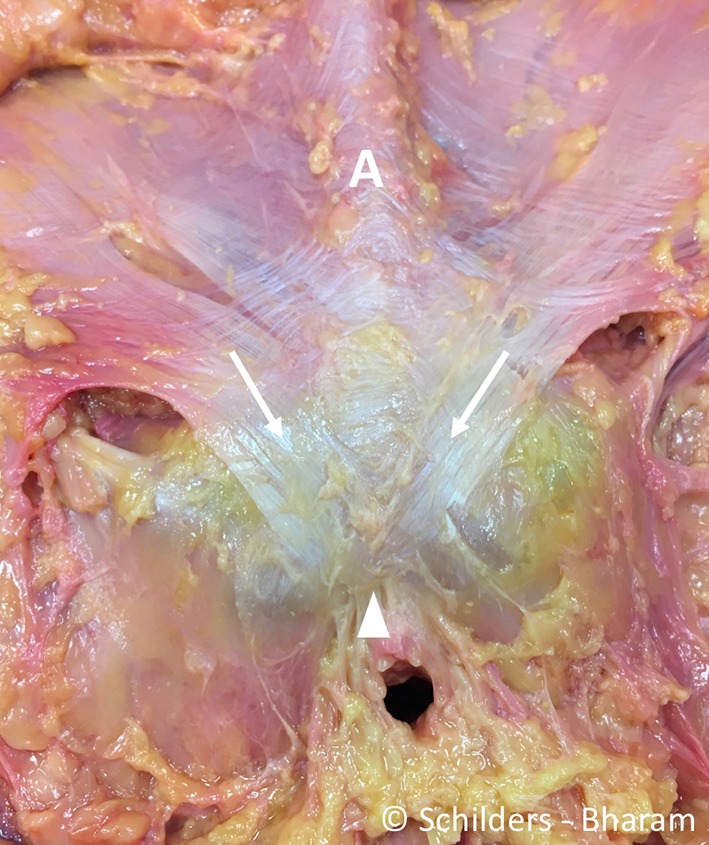
Male cadaver, after removal of the spermatic cords and the penis. There is a cord-like linea alba (*A*). The fibres of the external oblique aponeurosis that forms the medial part of the external inguinal ring (*arrow*) interlace with the superficial portion of the anterior pubic ligament (*arrowhead*)

The superficial and deep portions of the anterior pubic ligament interlace centrally over the deep portion of the anterior pubic ligament (Fig. 4a). In three of seven specimens, a cord-like variance to the linea alba was observed (Fig. 2).

**Fig. 3:**
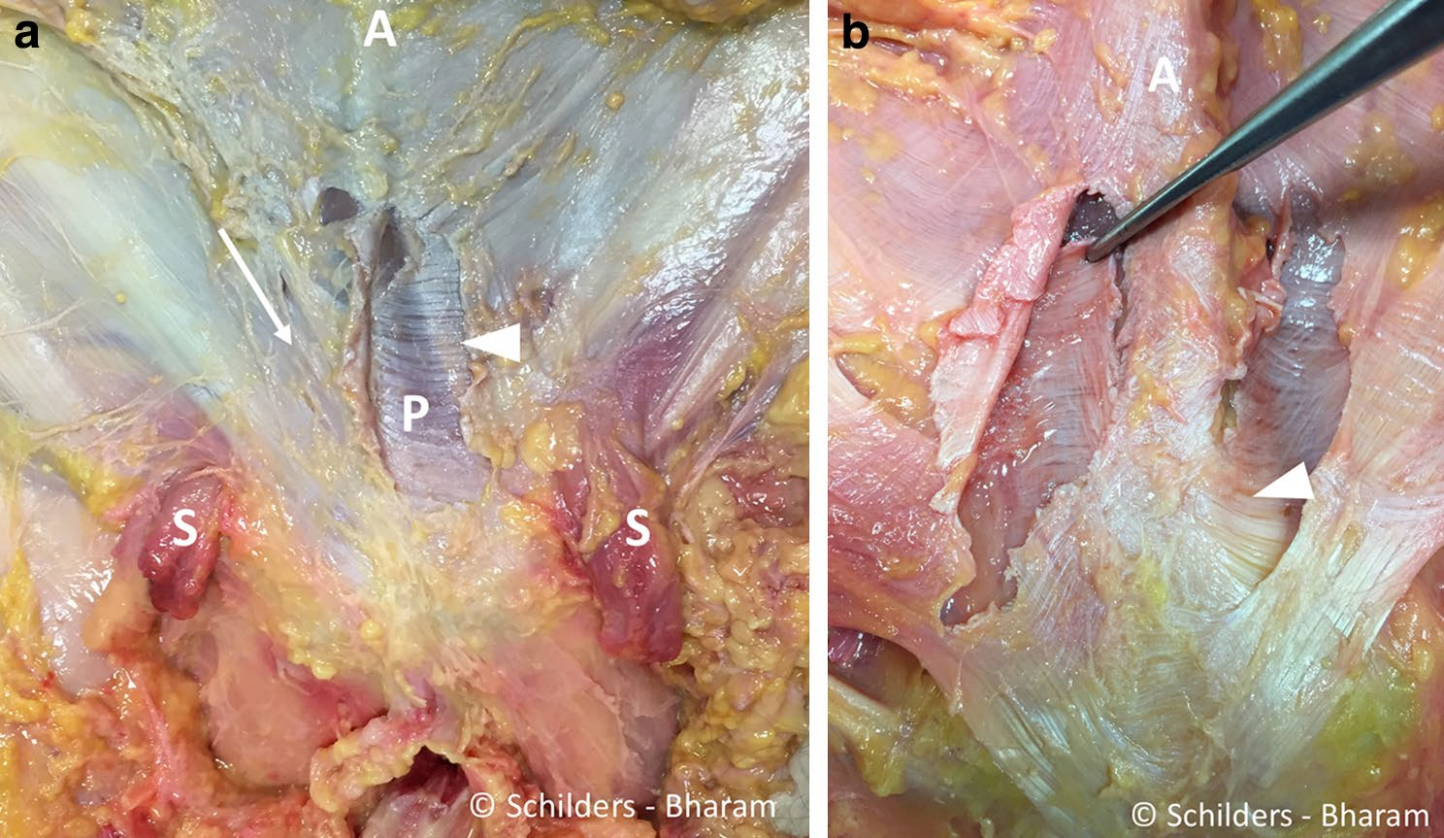
**a** Male cadaver, after a window was made in the anterior rectus fascia adjacent to the linea alba on the *left side*. The aponeurosis anterior to the pyramidalis muscle and the rectus abdominis comprises two layers. The superficial layer has oblique oriented fibres (*arrow right side*), and the fibres of the deeper layer have a more horizontal orientation. The window in the superficial fascia of the left abdominal external oblique muscle demonstrates the transverse orientation of the fibres of the deep layer (*arrowhead*), anterior to the pyramidalis muscle (*P*). *A* linea alba. *S* spermatic cord. **b** Forceps holding the deeper layer of the fascia anterior to the right pyramidalis muscle. Note the transversely oriented fibres. These fibres interlace with the fibres from the rectus abdominis aponeurosis and the linea alba (*arrowhead*). *A* cord-like linea alba

The aponeurosis anterior to the pyramidalis muscle and rectus abdominis is comprised of two layers (Fig. 3a, b). The superficial layer has oblique orientated fibres, the fibres of the deeper layer have a horizontal orientation and form the fascia anterior to the pyramidalis muscle, and both layers insert into the linea alba (Fig. 3b).The anterior pubic ligament is the medial anchor point for the part of the anterior abdominal aponeurosis medial to the external inguinal rings and pubic tubercles and fascia lata.

### After removal of the aponeuroses

#### Anterior pubic ligament (deep portion)

**Fig. 4:**
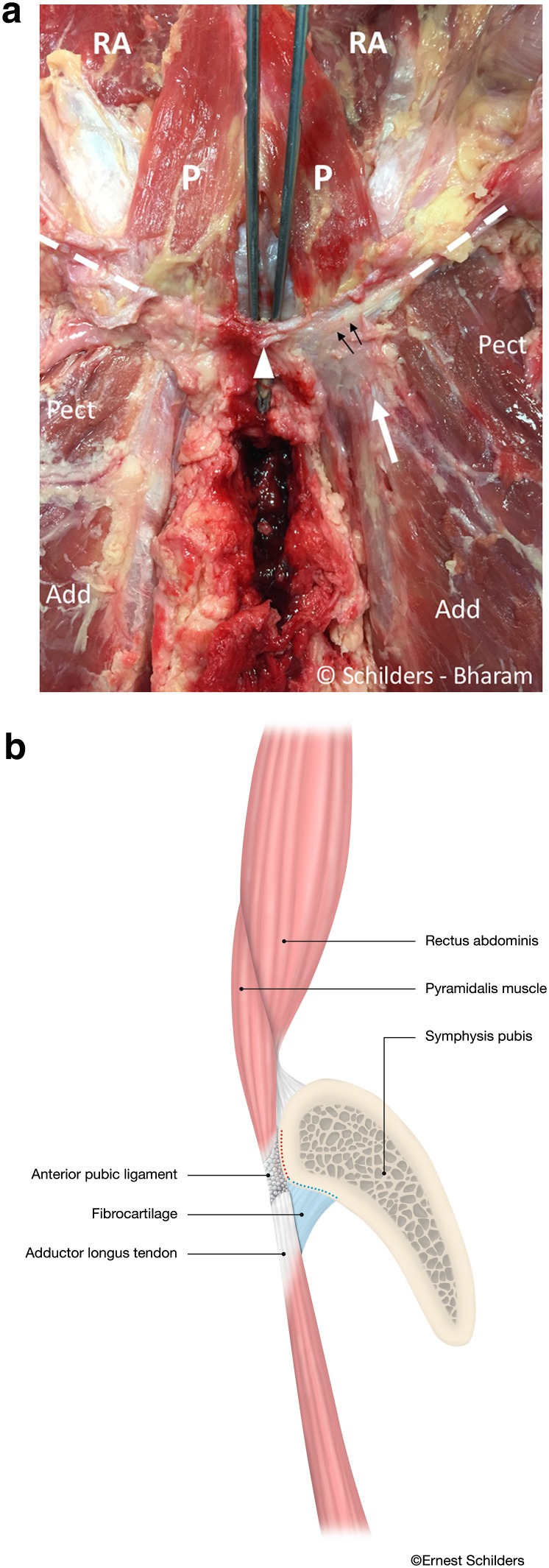
**a** Male cadaver. Anterior symphyseal area after removal of the entire aponeurosis demonstrates the remnants of the superficial portion of the anterior pubic ligament (*arrowhead*). The forceps is placed between under the anterior pubic ligament that spans over the symphyseal joint. The pyramidalis muscle (*P*) arises from the pubic crest and the anterior pubic ligament. The proximal adductor longus tendon (*white arrow*) interlaces with the deep portion of the anterior pubic ligament (*black arrows*). The anterior pubic ligament has transversely orientated fibres (*black arrows*) and extends into the inguinal ligament (*dashed line*) bilaterally. *Pect* pectineus muscle. *Add* adductor longus muscle. *RA* rectus abdominis muscle. **b** Sagittal section through the pubic bone medial to the pubic tubercles and lateral to the symphyseal joint. Drawing demonstrating the pyramidalis–anterior pubic ligament–adductor longus complex comprising the pyramidalis muscle (*P*), anterior pubic ligament (*arrow*) and adductor longus tendon (*arrow head*) and fibrocartilage (FC). The rectus abdominis (RA) is posterior to the pyramidalis muscle, and its musculotendinous junction cranial to the superior edge of the symphysis. With traumatic avulsion of the adductor longus, the fibrocartilage (*red dots*) of the adductor is avulsed in partial tears. Both the fibrocartilage (*red dots*) and anterior pubic ligament (*blue dots*) avulse in complete tears

The deep portion of the anterior pubic ligament is composed of transversely orientated fibres extending medially from the ipsilateral pubic tubercle across the symphysis to the pubic tubercle on the contralateral side. The ligament attaches to the pubic crest and crosses anteriorly to the symphyseal joint, connecting the pubic bones (Fig. 4a).

#### Pyramidalis muscle

**Fig. 5:**
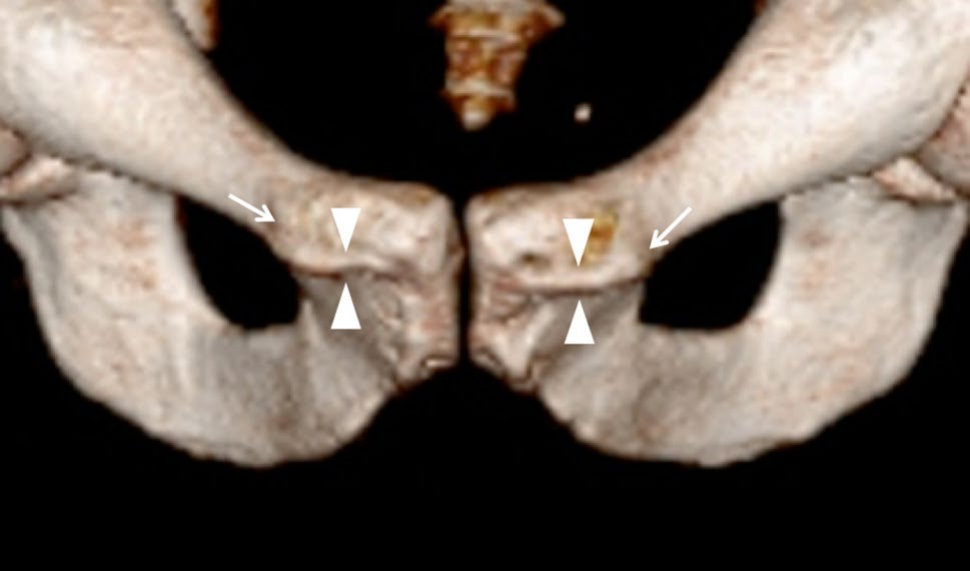
3D CT image of a male person showing the osteology of the anterior symphysis with the pubic crest (*arrowheads*) in between the pubic tubercles (*arrows*)

**Fig. 6:**
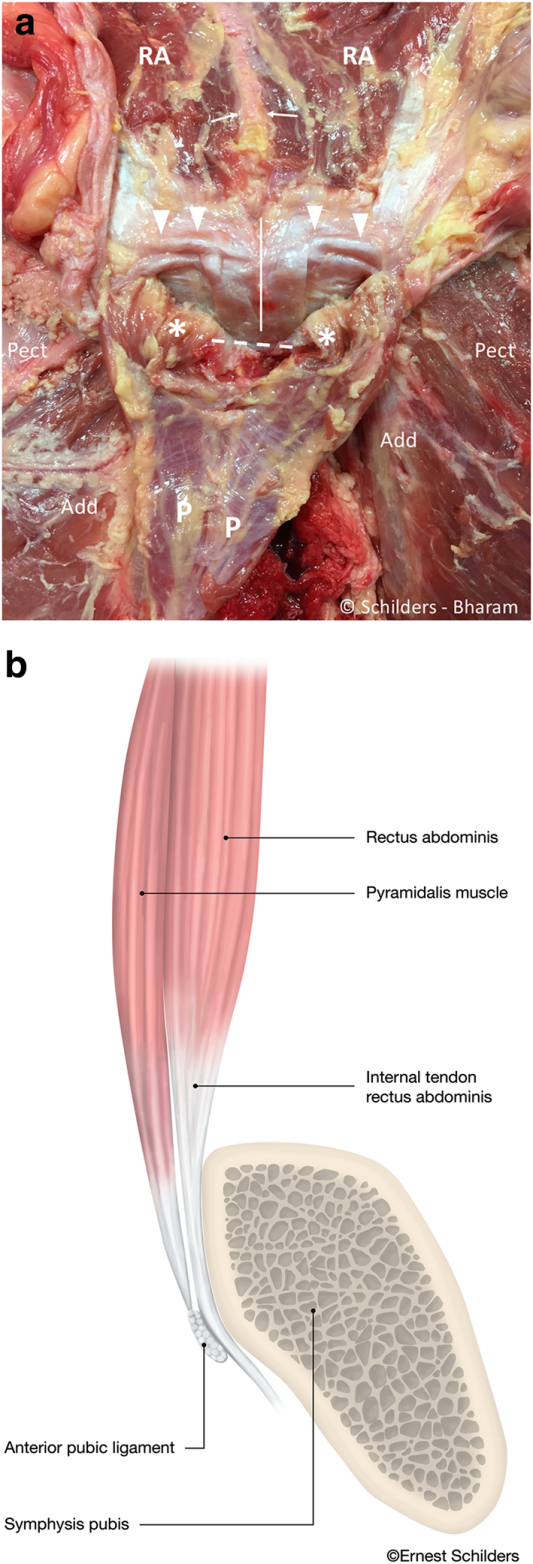
**a** Male cadaver, same specimen as Fig. 4. The pyramidalis muscle is sharply detached from the linea alba (*white arrows*) and folded distally. The internal tendons of the rectus abdominis run anterior to the symphyseal joint (*white line*), and the external tendon inserts on the superior lateral edge of the pubis (*arrowheads*). There is a thin aponeurosis on the posterior side of the pyramidalis muscle (P) with transverse fibre orientation. This aponeurosis does not cover the pyramidalis muscle anterior to the pubic bone (asterisk). *Pect* pectineus muscle. *Add* adductor longus muscle. *A* linea alba. *Dashed line* region of the anterior pubic ligament. *RA* rectus abdominis muscle. **b** Sagittal midline drawing demonstrating the anatomical relationship of the anterior pubic ligament (*arrowhead*), the pyramidalis muscle (*P*) and the internal tendon of the rectus abdominis (RA) at the level of the symphysis pubis. The internal tendon of the rectus abdominis receives contributions from *left* and *right sides* giving the tendon a Y-shape (*arrow*). The pyramidalis muscle inserts on the anterior pubic ligament, which spans the symphyseal joint and acts like a pulley through its position anterior to the internal tendon of the rectus abdominis muscle

A triangular pyramidalis muscle of the inferior abdominal wall on each side of the linea alba was observed in all specimens. The muscle arises from the pubic crest medial to the pubic tubercles (Fig. 5) and from the deep portion of the anterior pubic ligament by tendinous fibres, from which the muscular portion proceeds upwards and attaches to the linea alba. The muscle has no attachment to the anterior aspect of the pubic bone cranial to pubic crest. The width of the base extends from the pubic tubercle to the symphyseal midline, and no interspace was encountered between the bases of the pyramidalis muscles. The muscle is separated from the rectus abdominis by a thin, but well-defined aponeurosis (Fig. 6), which connects medially to the linea alba. The measurements of the anterior surface of the pyramidalis muscle are listed in Table 1.

**Table 1:** Measurement of the dimensions of the pyramidalis muscles: the range of measurements per specimen (between brackets) and average

Specimen	#1	#2	#3	#4	#5	#6	#7	Mean (SD)
Age at death (years)	75	64	79	65	74	60	67	69.1 (6.9)
Bissectrice Length: Left (cm)	7.0 (7.0–7.2)	8.8 (8.7–8.8)	7.9 (7.8–8.1)	7.9 (7.8–8.0)	7.4 (7.3–7.4)	8.6 (8.6–8.6)	8.3 (8.2–8.5)	8.0 (0.6)
Bissectrice Length: Right (cm)	8.7 (8.6–8.8)	8.7 (8.6–8.8)	7.9 (7.7–8.0)	7.9 (7.8–8.0)	8.4 (8.3–8.4)	8.3 (8.3–8.3)	8.3 (8.3–8.3)	8.3 (0.3)
Distal Width: Left (cm)	2.1 (2.0–2.1)	1.4 (1.4–1.4)	2.4 (2.4–2.4)	1.8 (1.7–1.9)	1.9 (1.8–1.9)	2.0 (2.0–2.0)	2.8 (2.7–2.8)	2.0 (0.4)
Distal Width: Right (cm)	2.1 (2.1–2.1)	1.6 (1.5–1.7)	2.3 (2.1–2.5)	1.5 (1.5–1.6)	1.9 (1.8–1.9)	1.7 (1.7–1.7)	2.9 (2.8–2.9)	2.0 (0.4)

The mean and standard deviation (st dev)

#### Adductor longus

The anterior tendon fibres interlace with the deep portion of the anterior pubic ligament (Fig. 4a, b)

**Fig. 7:**
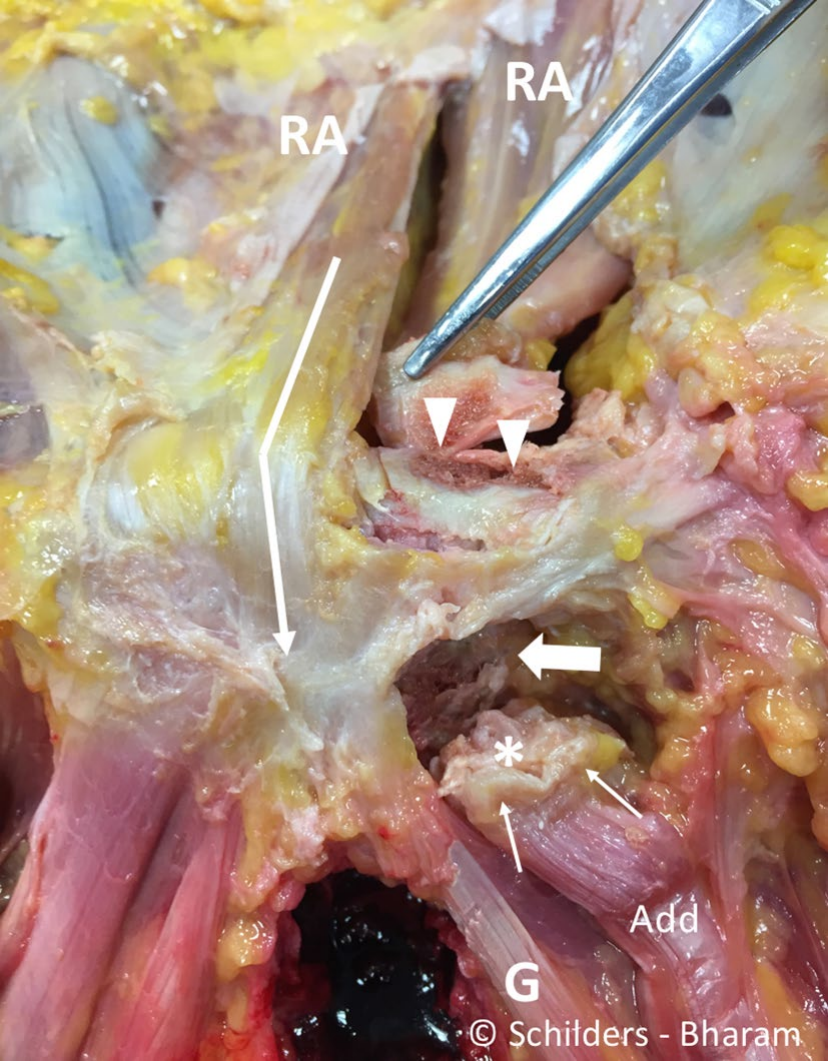
Male cadaver. It shows the anterior symphyseal and perisymphyseal area after resection of the pyramidalis muscle and the anterior pubic ligament. It demonstrates that the internal tendon of the rectus abdominis interlaces with the gracilis (*G*) and fascia lata (*arrow*). It shows the footprint of the external tendon of the rectus abdominis (*arrowheads*) and the footprint of the adductor longus fibrocartilage (*large arrow*). The adductor longus (Add) tendon (*thin arrows*) and the fibrocartilage (*asterisk*) are detached. *RA* rectus abdominis muscle

The adductor longus fibrocartilage deep to the tendon originates from the anterior pubic body inferior to the pubic crest (Figs. 4b, 7).

#### Rectus abdominis muscle

The rectus abdominis muscle arises from internal and external tendons, respectively (Fig. 6a). The external tendon is broader and attaches to the cranial border of the pubis between the pubic tubercle and the symphysis. The internal tendon is slender and interlaces with the contralateral internal tendon and descends below the deep portion of the pubic ligament [which seems to act similarly to a pulley (Fig. 6b)] to interlace distally with the fascia lata and gracilis (Fig. 7). Additionally, we observed that the musculotendinous junction of the rectus abdominis was proximal to the superior edge of the pubic bone in all specimens (Fig. 6a).

## Discussion

The most important finding of the present study is introduction of a new anatomical concept, i.e. the direct connection between the pyramidalis muscles and the adductor longus forming a pyramidalis–anterior pubic ligament–adductor longus complex (PLAC). In addition, it was observed that the only abdominal muscle anterior to the pubic bone is the pyramidalis and not the rectus abdominis.

To our knowledge, this study is the first to systematically examine the pyramidalis muscle and all its connections with the anterior pubic ligament, adductor longus enthesis and rectus abdominis muscle. A significant advantage of the study is that all dissections were performed on fresh-frozen cadavers, thereby avoiding potential confounders associated with cadaveric embalming such as tissue adhesions and shrinkage [10].

The study’s findings clearly contest the currently accepted concept that the adductor longus is connected with the rectus abdominis via an aponeurotic plate [14, 19, 20, 24] or that a fusion of the rectus abdominis with the adductor longus exists [11, 23]. The authors’ observation that rectus abdominis consists of a larger external tendon and a slender internal tendon is found in numerous anatomical textbooks [8, 9, 13, 15, 17, 29]. The current study demonstrated, similarly to other authors [8, 15–17, 26, 27], that the slender internal tendon of the rectus abdominis interlaces with that of the opposite side in the median line and runs only anterior to the symphyseal joint. A point of disagreement is the insertion of the internal tendon: some authors [15, 25] report it connects with the ligaments covering the symphysis pubis; our observations were that the internal tendon in males attaches to the fascia lata and gracilis and this is similar to findings of Schilders [27] and Schache [26].

The exact function of the internal tendon of rectus abdominis is unknown. The tendon runs under the deep portion of the anterior pubic ligament, which seems to act as a pulley system and prevents anterior displacement.

The present study also demonstrates that the aponeurosis that comprises the superficial layer, covering the lower abdomen, pubis and adductor area, has distinctly different connections than the deeper musculotendinous layer. Specifically, during our dissection we observed the aponeurosis of the external oblique and anterior rectus sheath to connect with the fascia lata over the adductor area via the anterior pubic ligament (superficial portion).

During the dissections, it was observed that there are two aponeurotic layers anterior to the pyramidalis muscles, identifiable as an obliqui and a transversalis aponeurosis, based on the observed orientation of each of the respective aponeurotic fibres. Additionally, we observed a thin aponeurosis, posterior to pyramidalis muscle, which separated it from rectus abdominis. This aponeurosis contains fibres with a distinct, transverse orientation, suggesting this structure most likely represents transversalis aponeurosis. Such transverse fibres can be readily observed during inguinal surgery. Most anatomical textbooks report that the aponeurosis anterior to pyramidalis and rectus abdominis comprises the obliqui and transversalis, but it is unclear as to whether or not the pyramidalis has a separate sheath [15, 33]. Hancock [16] and Ellis [13] report that the pyramidalis has it own fascial sheath, and imply that the muscle can contract independently of the recti muscles.

It has generally been assumed that the pyramidalis muscle is not large enough to generate a significant force load. However, a large variety in length (2.00–14.50 cm) and widths (0.50–6.00 cm) of the pyramidalis muscle has been reported and probably only larger variations of pyramidalis muscle are able to generate some force across the anterior abdomen [1, 2, 21].

Although it was not the aim of the study to report on anatomical variants, we found the pyramidalis was present bilaterally in all specimens examined. Other studies report that the muscle is often absent or double, or even triple on one or both sides [4, 8, 9, 13, 15, 16, 22, 29].

There is also a racial difference in the occurrence of the pyramidalis muscle; the pyramidalis has been noted to be absent in 20.3% of Caucasian, in 12.5% of African Americans, in Japanese around 3%, but is always present in Chinese people [2].

Several authors concur with our finding that the pyramidalis arises from the pubic crest and the deep portion of the anterior ligament [8, 13, 29]; others report it arises from the pubic crest, but do not report an attachment to the anterior pubic ligament [4, 9, 17, 22, 33]. There is consensus that the anterior pubic ligament consists of several superimposed layers and each layer possesses a different fibre orientation. Specifically, the superficial fibres pass obliquely, while the fibres of the deep portion contain a transverse orientation [3, 9, 15, 17, 30]. To our knowledge, none of the more recent anatomy studies mention the presence of the anterior pubic ligament [5, 11, 27, 30].

The adductor longus attaches to the inferior part of the pubic crest and the deep portion of the anterior pubic ligament and contains a fibrocartilaginous enthesis [11, 27, 28]. We have observed during surgery that the adductor longus fibrocartilage has a pyramidal shape and a triangular base and is positioned between the adductor tendon and the pubic crest (Fig. 4b). The fibrocartilage is easily recognized during surgery, with a reported bony footprint measuring 1.5 cm by 1.9–2.5 cm [11, 12]. We have also observed, both on MRI and intraoperatively, that with traumatic avulsions of the adductor longus the fibrocartilage is always at least partially if not completely detached.

It has been the first author’s observation that with traumatic adductor longus avulsions from the anterior pubic bone the end-to-end connection between pyramidalis muscle and adductor longus often remains intact. This might give the impression during surgery that the adductor tendon/pyramidalis complex is intact; however, with inspection under the tendon a separation from the pubic bone can be observed. Accordingly, this strong connection demonstrates the presence of a pyramidalis–anterior pubic ligament–adductor longus complex (PLAC). In our experience, lateral displacement of this complex only occurs with a disruption of the deep portion of the anterior pubic ligament which lies medial to the PLAC. A post-traumatic partial avulsion of the fibrocartilage of the adductor longus is also common; here, we have observed an intact adductor longus tendon, pyramidalis muscle and anterior pubic ligament.

The findings of the present study also contain clear radiological implications. With regard to the position of the pyramidalis, on sagittal MRI views the only abdominal muscle directly anterior to the pubic bone is the pyramidalis with the internal tendon of rectus abdominis only visualized at the direct midline. However, when moving laterally in either direction, it is fibres of the pyramidalis muscle, and not the rectus, that are visualized. On such non-midline views, the muscle belly of the rectus abdominis is never anterior to the pubic bone and always proximal to the superior edge of the symphysis. Thus, the PLAC should be readily observed in all non-midline sagittal imaging between the pubic tubercles (Fig. 4b), while the internal tendon of the rectus is only visualized at the direct midline (Fig. 6b).

The present study is not without limitations. Although the study only involved seven male cadavers, our findings were consistent. The dissections were performed by two surgeons with extensive soft tissue experience, but the study could have benefited from additional histological examination of the anatomical connections.

The authors believe this study’s findings of a direct connection between the pyramidalis and adductor longus muscle as well as the identification of a pyramidalis–anterior pubic ligament–adductor longus complex (PLAC) to be invaluable in the management of proximal adductor avulsion. A clear understanding of the anatomical connections as highlighted by this study is crucial both for the interpretation of the MRI images and intraoperatively, as such familiarity will allow for recognition of the anatomical connections of the PLAC and ultimately achieve anatomical repairs following acute proximal adductor avulsions. All athletes with a clinical presentation consistent with an acute adductor avulsion should be sent for an MRI to study the injury pattern and determine the severity of the injury.

## Conclusions

The study identifies the pyramidalis as the only muscle anterior to the pubis.

The rectus abdominis is not connected to the adductor longus. The anterior pubic ligament is an important anchor point for both the superficial aponeurotic layers and the deep musculotendinous layer.

Both the adductor longus and pyramidalis muscle attach to the anterior pubic ligament. The pyramidalis muscle has a strong direct end-to-end anatomical connection with the adductor longus tendon (presence of a pyramidalis–anterior pubic ligament–adductor longus complex).

The adductor longus origin is stabilised by the anterior pubic ligament and the fibrocartilage attachment into the pubic bone.

The anatomical findings are important for imaging interpretation, surgical management and future understanding of injury patterns associated with proximal adductor longus injuries.
